# Varied Presentation of Fulminant Blastomycosis with Prostatitis and Acute Respiratory Distress Syndrome in a Patient With High Inoculum Inhalation: A Review of Diagnosis and Management

**DOI:** 10.7759/cureus.9686

**Published:** 2020-08-12

**Authors:** Shyam kiran Gandam Venkata, Joel Gieswein, Sai Sruthi Bhuram

**Affiliations:** 1 Critical Care Medicine, Springfield Clinic/Southern Illinois University (SIU) School of Medicine, Springfield, USA; 2 Emergency Medicine, Southern Illinois University (SIU) School of Medicine, Springfield, USA; 3 Internal Medicine, SVS Medical College, Hyderabad, IND

**Keywords:** fungal lung infection, endemic infections, pulmonary blastomycosis, fungal prostatitis, ards (acute respiratory distress syndrome), blastomyces dermatitidis

## Abstract

Blastomycosis is an uncommon granulomatous disease caused by infection with thermally dimorphic fungi of the genus Blastomyces. Although pulmonary infections from Blastomyces dermatitidis are uncommon, it is important to understand the geographical distribution, presentation, diagnosis, and management of treating this condition. We report a case of fulminant blastomycosis after high inoculum inhalation, with involvement of the prostate on presentation, which progressed to acute respiratory distress syndrome.

## Introduction

Acute respiratory distress syndrome (ARDS) is a very deadly condition. *Blastomyces* dermatitidis is an uncommon pathogen to cause ARDS, with an exceedingly high mortality rate. Most (96.7%) of the blastomycosis-related deaths occurred in the southern and midwestern regions, and a small proportion of deaths occurred in the northeastern and western regions [1]. The endemic fungi are more widespread in their locale than realized by local practitioners, which can cause virulent disease in patients regardless of immune status. The dimorphic fungi draw on several strategies to evade innate and adaptive immunity [2]. We describe here a case of *Blastomyces dermatitidis* in a healthy middle-aged man with minimal comorbid conditions from central Illinois who was induced by high inoculum inhalation, leading to prostatitis with disseminated infection and subsequent mortality from ARDS. We believe that this is one of the very few cases of disseminated *Blastomyces *with confirmed prostatitis and ARDS currently present in the literature. The following case report describes different aspects regarding reliability of diagnostic modalities, the importance of history, presentation, and epidemiology.

## Case presentation

A 68-year-old male from central Illinois was admitted by the urology service for acute urinary retention and pain around his bladder catheter, which was placed three days prior at a urology clinic for the same complaint and was started on a course of ciprofloxacin. Medical history is significant for benign prostatic hyperplasia with no urinary retention symptoms in the past, hypertension, hyperlipidemia, and prostate cancer under active surveillance. The patient is an avid hunter, with a recent history of having moved several tons of soil in his backyard approximately two weeks prior to hospital admission.

**Figure 1:**
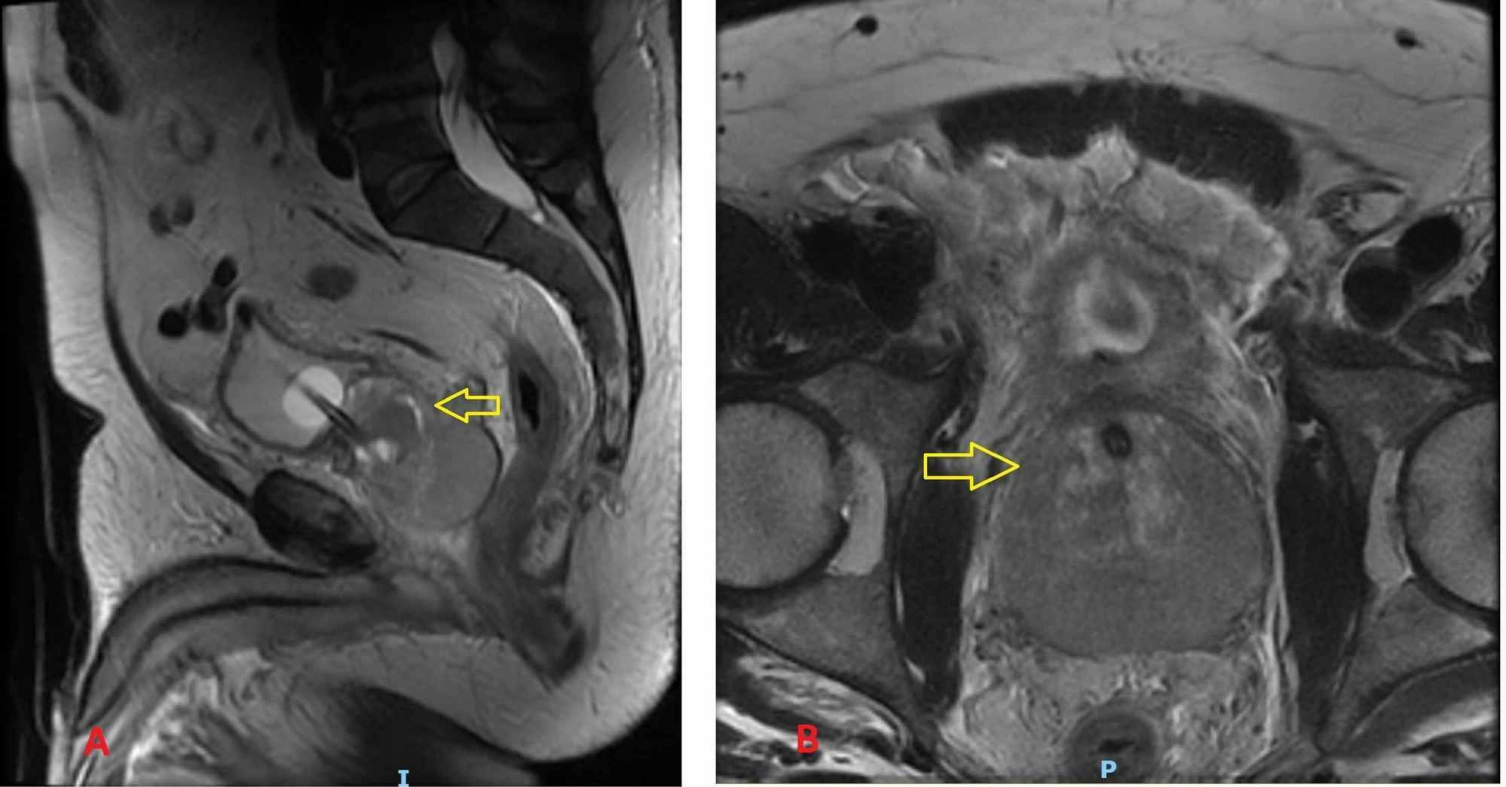
Sagittal (A) and axial (B) T2 MRI images showing marked enlargement of the prostate gland, most notably the peripheral zone with marked T2 hypointensity. Findings were concerning for prostatitis.

Initial urologic workup after admission revealed prostatitis discovered on MRI (Figure 1). Three days after in-patient admission, he developed a fever and shortness of breath. Multiple cultures were obtained, as well as other blood tests, urinalysis, and urine culture. Chest X-ray performed day after admission showed bilateral infiltrates. The patient's antibiotic coverage was broadened and transferred to the intensive care unit for worsening hypoxemia and respiratory distress. He eventually developed ARDS requiring intubation and mechanical ventilation with lung protective strategies. Initial urine culture showed sterile pyuria with no organism growth, with no organisms seen on gram stain. Steroids were initiated, and a bronchoalveolar lavage (BAL) was performed shortly after intubation, which did not demonstrate any bacterial or viral infection on initial culture report. Gram stain of this fluid did show neutrophilia but with a low absolute volume of neutrophils. After three days of lavage, culture eventually showed yeast, which grew *Blastomyces dermatitidis*. Serum tests for *Histoplasma capsulatum* and *Blastomyces dermatitidis* were negative, as was testing for other fungi. The high inoculum exposure to soil prior to admission is presumed as the possible source of *Blastomyces dermatitidis*. His clinical condition progressively worsened to multiorgan failure requiring renal replacement therapy. Considering worsening P/F ratios from moderate ARDS of 164 to severe ARDS of 94, the patient was considered for extracorporeal membrane oxygenation (ECMO) capabilities by the regional ECMO center. The patient expired prior to being placed on ECMO.

## Discussion

Clinical guidelines from the Infectious Disease Society of America (IDSA) published in 2008 recommend diagnosing blastomycosis from sputum samples through visualization or other staining methods when rapid diagnosis is necessary. The IDSA does not recommend immunofixation techniques or other enzyme assays due to low sensitivity and specificity [3]. A study from the Journal of Clinical Microbiology found the sensitivity of these assays in pulmonary blastomycosis to only be 64.3% in the serum, 82.7% in urine, and 62.5% from BAL sampling. Sensitivities were lower in other forms of blastomycosis. The authors conclude that antigen testing alone should not exclude the diagnosis of blastomycosis [4].

*Blastomyces dermatitidis* is a rare cause of infection, and one paper cited an incidence of four patients needing antifungal treatment per 100,000 persons in endemic areas [5]. There are several case reports in the literature of pulmonary blastomycosis leading to ARDS with significant mortality rates [6,7]. In one of the largest case series of *Blastomyces* infection from the state of Indiana, 15% of their patients developed ARDS [8]. In a case series from Mississippi, only 8% of patients developed ARDS [7]. ARDS associated with *Blastomyces* infection is extremely lethal, with a mortality rate of 50-89% despite treatment [9]. Interestingly, unlike traditional teaching in medical schools, many patients who develop blastomycosis are immunocompetent [5,6], with only 16% being immunosuppressed in the Indiana case series [8]. In the state of Illinois, where we practice and where our patient resided, 500 cases of *Blastomyces* infection were analyzed over a 10-year period. Only 7% of the patients died in this study, with risk factors for death being African American race, age greater than 65 years, and greater than 128 days from onset of symptoms to diagnosis [10].

**Figure 2:**
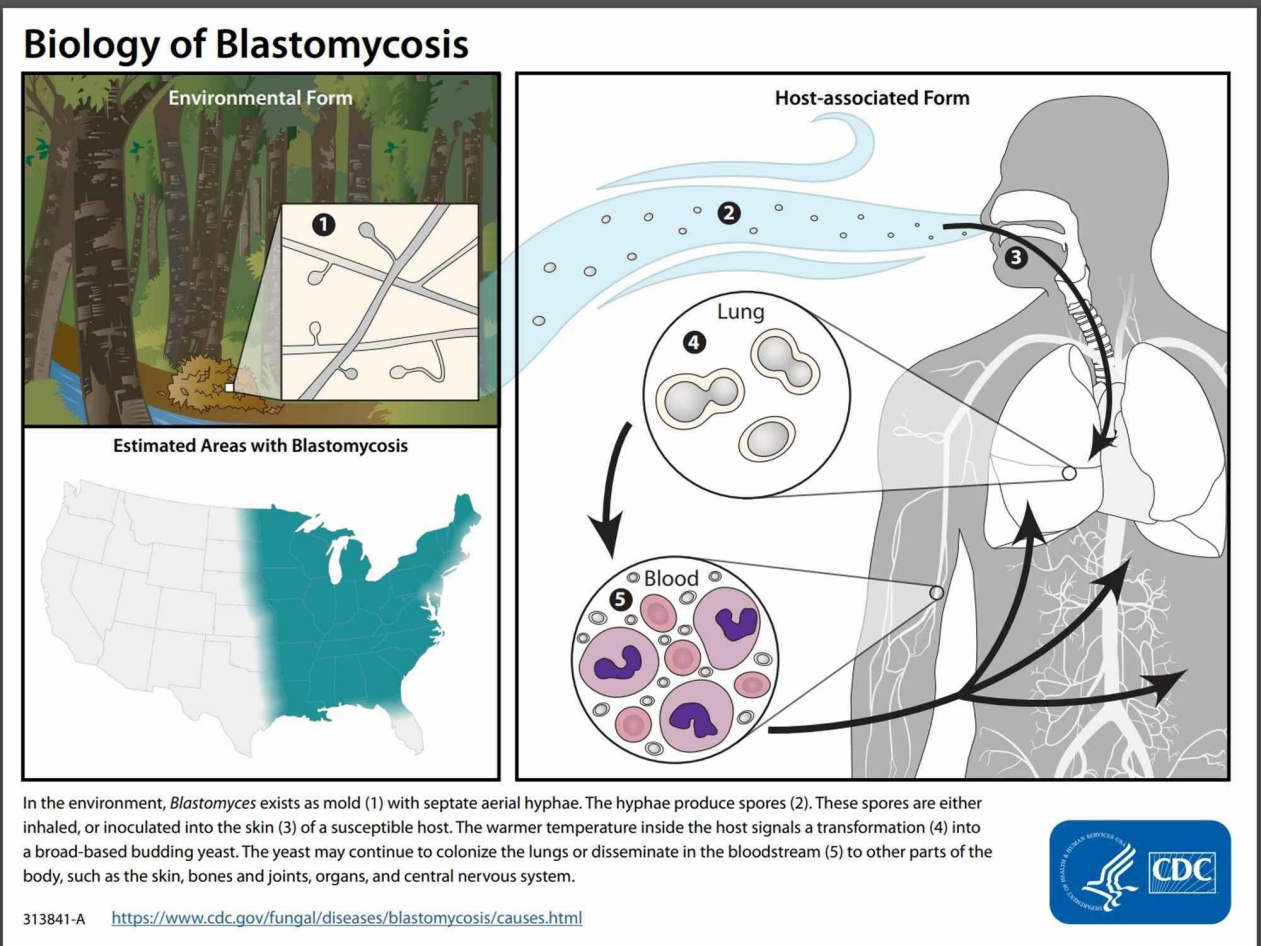
Centers for Disease Control figure outlining biology of infection and areas at highest risk for Blastomyces infections

*Blastomyces* lives in the environment, particularly in moist soil and in decomposing organic matter such as wood and leaves. In the United States, the fungus mainly lives in the midwestern, south-central, and southeastern states, particularly in areas surrounding the Ohio and Mississippi River valleys, the Great Lakes, and the Saint Lawrence River (Figure 2). Usual mechanism of infection with *Blastomyces* is by inhalation of its conidia. These conidia then germinate and infect the local pulmonary tissue or occasionally cause systemic infection. Pulmonary infections are often asymptomatic, frequently attributed to be a common cold [9]. After pulmonary infections, skin is the second most common site of infection. Genitourinary infection with *Blastomyces* is less common than bone, skin, or lung but occurs in up to 30% of patients with systemic blastomycosis [11].

Treatment of blastomycosis is with amphotericin B in moderate-to-severe disease or with itraconazole in mild disease [11]. Corticosteroids were shown to be of benefit in a small case series of two patients in the Chest journal [12]. ECMO has been shown to be of benefit in some patients with ARDS induced by *Blastomyces* with refractory hypoxemia [13]. When starting amphotericin B, there is a significant concern among clinicians regarding the potential for renal failure. A study performed by the American Society of Microbiology found that the liposomal formulations of amphotericin B were significantly less nephrotoxic than amphotericin B deoxycholate, without sacrificing efficacy [14].

Our patient appeared to have inhaled a large inoculum of *Blastomyces dermatitidis* conidia during his yardwork. The *Blastomyces* then seemed to settle into our patient’s prostate due to his underlying prostatic disease from disseminated spread. His first symptoms were urinary retention, which was subsequently determined to be prostatitis with sterile pyuria. Most case reports and case series reported a pulmonary source of infection with *Blastomyces* that led to ARDS [5,6,8,11]. Our patient had a positive BAL culture result. In a review of Wisconsin cases, tracheal aspirates readily showed the broad-based budding yeast pattern of *Blastomyces* [6], while the lavage fluid from our patient did not clearly demonstrate this pattern. *Blastomyces*-related infections require evaluation from the ground up with a good history and high index of suspicion in endemic areas.

## Conclusions

*Blastomyces*, although rare, can cause infection in immunocompetent and immunocompromised patients alike. A review of the Centers for Disease Control and Prevention guidelines in understanding the geographical distribution of these endemic mycoses is important for a physician to keep these presentations in their differential diagnosis. For a patient presenting with fulminant pneumonia and/or ARDS, clinicians should consider initiating anti-fungal therapy if collateral history demonstrates a possible exposure to *Blastomyces *in consultation with infectious diseases team early in the disease course. Diagnosing fungal pneumonia remains a diagnostic challenge. Immunodiffusion assays for fungi have poor sensitivity and specificity and should not be relied upon for treatment decisions. Rather, allow patient’s history and response to treatment to guide decision-making.
